# DPAM-AI: a domain parser for AlphaFold models powered by artificial intelligence

**DOI:** 10.1093/bioinformatics/btae740

**Published:** 2024-12-13

**Authors:** Jesse Durham, Jing Zhang, Richard D Schaeffer, Qian Cong

**Affiliations:** Eugene McDermott Center for Human Growth and Development, University of Texas Southwestern Medical Center, Dallas, TX 75390, United States; Harold C. Simmons Comprehensive Cancer Center, University of Texas Southwestern Medical Center, Dallas, TX 75390, United States; Department of Biophysics, University of Texas Southwestern Medical Center, Dallas, TX 75390, United States; Eugene McDermott Center for Human Growth and Development, University of Texas Southwestern Medical Center, Dallas, TX 75390, United States; Harold C. Simmons Comprehensive Cancer Center, University of Texas Southwestern Medical Center, Dallas, TX 75390, United States; Department of Biophysics, University of Texas Southwestern Medical Center, Dallas, TX 75390, United States; Department of Biophysics, University of Texas Southwestern Medical Center, Dallas, TX 75390, United States; Department of Biochemistry, University of Texas Southwestern Medical Center, Dallas, TX 75390, United States; Eugene McDermott Center for Human Growth and Development, University of Texas Southwestern Medical Center, Dallas, TX 75390, United States; Harold C. Simmons Comprehensive Cancer Center, University of Texas Southwestern Medical Center, Dallas, TX 75390, United States; Department of Biophysics, University of Texas Southwestern Medical Center, Dallas, TX 75390, United States

## Abstract

**Motivation:**

Due to the breakthrough in protein structure prediction by AlphaFold, the scientific community has access to 200 million predicted protein structures with near-atomic accuracy from the AlphaFold protein structure DataBase (AFDB), covering nearly the entire protein universe. Segmenting these models into domains and classifying them into an evolutionary hierarchy hold tremendous potential for unraveling essential insights into protein function.

**Results:**

We introduce DPAM-AI, a Domain Parser for AlphaFold Models based on Artificial Intelligence. DPAM-AI utilizes a convolutional neural network trained with previously classified domains in the Evolutionary Classification Of protein Domains (ECOD) database. DPAM-AI integrates inter-residue distances, predicted aligned errors, and sequence and structural alignments to previously classified domains detected via sequence (HHsuite) and structural (Dali) similarity searches. DPAM-AI has demonstrated its power through rigorous tests, excelling in several benchmark sets compared to its predecessor, DPAM, and other recently published domain parsers, Merizo and Chainsaw. We applied DPAM-AI to representative AFDB models for proteins classified in Pfam. We obtained representative 3D structures for 18 487 (89%) of the 20 795 Pfam families. The remaining families either (i) belong to viral proteins that were excluded from AFDB or (ii) do not adopt globular 3D structures. Our structure-aware domain delineation uncovered a considerable fraction (15%) of Pfam domains containing multiple structural and evolutionary units and refined the boundaries for over half.

**Availability and implementation:**

Pfam and corresponding DPAM-AI domains are at http://prodata.swmed.edu/DPAM-pfam/. Our code is deposited at https://github.com/Jsauce5p/DPAM/tree/dpam_ai, and updates will be released through https://github.com/CongLabCode/DPAM.

## 1 Introduction

AlphaFold2 (Jumper *et al.* 2021a), released by DeepMind in 2020, was the first computational method able to predict 3D structures of proteins to near-atomic accuracy, including numerous difficult examples of fast-evolving proteins (Jumper *et al.* 2021b). DeepMind has used AlphaFold to model over 200 million proteins, including the entire proteomes of humans and many other organisms relevant to public health and research, and released these models via the AlphaFold protein structure database (AFDB) (Varadi *et al.* 2022). Protein structures are valuable for function prediction and evolutionary studies (Whisstock and Lesk 2003, Worth *et al.* 2009). The similarity between 3D structures is retained at a much greater evolutionary distance than sequences, allowing us to find evolutionary links between distant homologs.

We need new methods to process this large number of predicted structures and gain functional insights. Partitioning these AlphaFold models into domains, i.e. the evolutionary, structural, and functional units of proteins, is an essential step toward making sense of such data. Various methods (Holm and Sander 1994, Alexandrov and Shindyalov 2003) have been developed to partition experimentally determined 3D structures into domains based on their structural properties, i.e. they can form globular, compact structures. However, domain parsing by physical properties alone does not always produce satisfactory results because multiple evolutionary units might be tightly packed together in some proteins (Holm 2019).

Detecting homology through sequence and structure similarities (Dietmann and Holm 2001, Steinegger *et al.* 2019, Liu *et al.* 2024) to previously classified domains in databases is another effective way to parse domains (Buljan and Bateman 2009). Databases for domain classifications can be broadly divided into sequence-based and structure-based. For example, the Pfam database (Sonnhammer *et al.* 1998) is a widely used domain classification resource, where domains are recognized by aligning sequences of different proteins and identifying homologous regions. Sequence-based classifications (Marsolo and Parthasarathy 2008) are frequently able to cover the entire protein universe but fall short of precisely defining domain boundaries (Galzitskaya and Melnik 2003). In contrast, structure-based classifications, such as CATH (Class, Architecture, Topology, Homologous superfamilies) (Sillitoe *et al.* 2021) and the Evolutionary Classification Of Domains (ECOD) (Cheng *et al.* 2014), can accurately determine boundaries between domains using 3D structural information but were previously limited to proteins with experimentally determined 3D structures in the Protein Data Bank (PDB). The similarity between 3D structures can detect homology at distances where sequence conservation is hardly detectable. Thus, structural comparisons allow these structure-based classifications to detect remote homology and classify proteins that have evolved beyond recognition by sequence (Vogel *et al.* 2004).

AlphaFold models for 200 million proteins have transformed modern structure biology (Pei *et al.* 2022). This breakthrough will chart protein science by facilitating the characterization of protein function in the coming decade (Durham *et al.* 2023). One approach to mining this massive collection of structural data and gaining biological insights is identifying and classifying domains in these predicted structures into evolutionary hierarchies. Homology codified through this classification process will guide future functional inference and experimental characterization of new proteins.

Compared to experimentally determined 3D structures, AlphaFold models contain more flexible linkers, disordered regions, and poorly modeled regions. Identifying globular domains from these models is the first step toward classifying them and gaining functional insights. To meet this need, we previously developed a Domain parser of AlphaFold models (DPAM) to recognize and delineate globular domains from these models using probabilities of two residues belonging to the same domain. Such probabilities were derived from inter-residue distances in 3D structures, predicted aligned errors (PAEs), and ECOD domains found by sequence (HHsuite) and structural (Dali) similarity searches. DPAM was benchmarked on ECOD domains and has been used to expand ECOD to cover AlphaFold models (Schaeffer *et al.* 2023, Zhang *et al.* 2023).

Several other domain parsers designed for AlphaFold models became available after the initial release of DPAM, such as Merizo (Lau *et al.* 2023) and Chainsaw (Wells 2023), both of which use artificial intelligence (AI) to analyze AlphaFold models and are trained using CATH domains, showing superior performance over traditional domain parsers based on structural properties. Merizo and Chainsaw did not use homology-based evidence. However, homology-based evidence will allow us to partition tightly packed evolutionary units and increase the consistency between newly detected and previously classified domains in a target database, facilitating downstream domain classification.

Using AI to integrate structural features and homology-based evidence, we introduce DPAM-AI, a convolutional neural network (CNN) trained on 18k AlphaFold models containing 28k ECOD domains. DPAM-AI outperformed DPAM, Merizo, and Chainsaw on various benchmarks, regardless of whether we use ECOD or CATH domains as the ground truth. We applied DPAM-AI to representative proteins classified in Pfam, allowing us to identify the structure-based domains corresponding to each Pfam family. We share our results at http://prodata.swmed.edu/DPAM-pfam/, providing 3D domains for 90% of families and suggesting structure-based boundaries for them. In the future, DPAM-AI will be used to parse AlphaFold models into domains, streamlining their assignment into ECOD. Expansion of ECOD to include AlphaFold models will reveal new insights into domain evolution and protein function.

## 2 Materials and methods

### 2.1 Preparing the training and testing datasets

The training dataset for DPAM-AI is the same as DPAM, which was derived from ∼1 million AlphaFold models initially released via AFDB. This training set consists of 18 759 representative AlphaFold models with closely related (identity >95%) ECOD-classified (Schaeffer *et al.* 2018) PDB entries. AFDB has released ∼200 million models, allowing us to find additional AlphaFold models with corresponding ECOD or CATH entries for model validation. We obtained 54 638 AlphaFold models satisfying this criterion based on the mapping of Uniprot entries to PDB (https://ftp.uniprot.org/pub/databases/uniprot/current_release/knowledgebase/idmapping/). We removed AlphaFold models homologous to the training dataset detectable by BLASTP (e-value <0.00001), resulting in an independent test set of 3680 AlphaFold models. Proteins (3437 and 2468) from the test set were previously classified in the ECOD and CATH databases, respectively, and used to generate test sets. We clustered the proteins in both test sets by MMseqs (Steinegger and Söding 2017) (identity: 50, coverage: 80) and selected one representative per cluster, resulting in 3075 and 2249 proteins in the ECOD and CATH test sets, respectively.

### 2.2 Gathering homology-based evidence for domain parsing

The inputs of DPAM-AI include similar ECOD domains found by HHsuite and Dali. To evaluate the performance of DPAM-AI on the CATH test set, we used CATH domains as homology-based evidence. To avoid simplifying the task by finding a highly similar ECOD (CATH) domain, we detected each input protein’s closely related ECOD (CATH) domains by BLAST (e-value <0.00001) (Camacho *et al.* 2009). We removed such close homologs from the HHsuite and Dali hits.

We downloaded the PDB70 database from HHsuite GitHub and derived an ECOD70 set with 63 065 ECOD domains based on PDB70. Similarly, we prepared a CATH70 set with 50 549 domains. For each input protein, we identified its HHsuite hits in the PDB70 database. Based on definitions of ECOD (CATH) domains, we partitioned each PDB70 hit into ECOD (CATH) domains. We identified the structural hits for each input protein by Foldseek (van Kempen *et al.* 2024) (-e 1000—max-seqs 1000000) against the ECOD70 (CATH70) database. We used Foldseek with a relaxed cutoff as a fast tool to find candidates for the slower but more sensitive tool, Dali. Dali was used to align HHsuite and Foldseek hits to the query. Dali aligns an ECOD (CATH) domain to only one segment in a query structure, even if the query contains multiple copies of this domain. To alleviate this problem, we developed an iterative Dali alignment procedure: in iterations, the segment of a query aligned to a hit in a previous round was excluded, and the remaining structure was used to perform a Dali search until no similarity was found between the remaining portion of the query and the hit.

### 2.3 Generating input matrices for the DPAM-AI network

DPAM-AI takes a series of 2D matrices in the shape of (500, 500) as inputs. For proteins of <500 residues, we added x extra residues on the left and (500-L-x) extra residue on the right, where x is a random number between 0 and 500-L. Values in the input matrices involving the extra residues were filled with 0. For proteins of >500 residues, we took n 500-residue crops with the following rules:

n=1, if L≤750;

n=2, if 750<L≤1000;

n=3, if 1000<L≤1500;

n=4, if 1500<L≤2000;

n=5, if L>2000.

Each 500-residue crop contains one or two segments in sequence, which was determined randomly. Using crops of two segments, i.e. discontinuous crops, allows the model to learn to handle residue pairs that are >500 residues apart. The start and end of each segment were also determined randomly. The above padding or cropping procedure was repeated for every epoch in the training using independently determined random numbers, which resulted in slightly different training datasets for each epoch to reduce the chance of over-training.

We used the following 2D matrices as inputs: one distance matrix, one PAE matrix, two residue position matrices, ≤10 HHsuite matrices, and ≤10 Dali matrices. The distance matrix reflects the minimal distance between the heavy atoms of each pair of residues, while the PAE matrix is downloaded from AFDB. Each element in a 2D matrix represents the property of a pair of residues, i and j, and these numbers were stored in two residue position matrices. We identified “acceptable HHsuite hits” and “acceptable Dali hits” based on criteria described in our DPAM paper (Zhang *et al.* 2023). These hits are not necessarily homologous to the query but might assist domain parsing. We ranked the acceptable HHsuite hits by HHsuite probability (HHprob), and we generated a 2D matrix for each hit where position (i, j) was filled with the HHprob if they were both aligned to this hit; if not, the element is set to 0. Based on the mapping to query residues of each hit, we grouped the HHsuite hits into groups. Starting from the top of the ranked list of hits, we merged an additional hit into an existing group if its overlap with other hits in the group is less than 20% of the query residues covered by the current hit or by other hits in the group collectively; if a hit could not be merged into any existing group, we started a new group with this hit. We merged the 2D matrices for hits in the same group and took the maximal value at each position. We converted the Dali hits into 2D matrices similarly, except for replacing HHprob with Dali Z-score. We allowed up to 10 matrices from HHsuite and Dali hits, respectively, prioritizing hits showing higher confidence (HHprob and Dali Z-scores).

### 2.4 Training and application of the DPAM-AI network

We identified close homologs among the training dataset by BLASTP (e-value <0.00001 and identity >30%) and grouped proteins into clusters. We used the training dataset to train DPAM-AI, leaving a random sample of 5% of the training clusters as a validation set to monitor the performance while training. The DPAM-AI network outputs a 2D matrix with predicted probabilities for each pair of residues to be in the same domain. The binary cross entropy (BCE) loss function was used to compare the prediction and the 2D representation of ECOD domain definitions (residue pairs in the exact domains marked as 1 and other pairs as 0). DPAM-AI was trained for 30 epochs to minimize the BCE loss until the validation loss reached a plateau.

To apply the DPAM-AI network to a protein with L residues, we converted the input 2D matrices with the shape of (L, L) to crops of the shape (500, 500). Similarly to how we processed the training data (see above), we padded proteins with <500 residues and systematically sampled continuous and discontinuous crops (made of two segments in the sequence) to cover the entire input matrices. If a residue pair (i, j) is included in multiple (500, 500) crops, we averaged the model outputs from these crops.

### 2.5 Converting the DPAM-AI network outputs to domain definitions

The output of DPAM-AI is a 2D matrix with predicted “same-domain” probabilities for residue pairs. We converted this matrix to domain definitions using a similar pipeline implemented in DPAM (Zhang *et al.* 2023). We detected disordered regions as segments showing high PAE values against the rest of a protein. For each residue, we identified its “PAE neighbors” as other residues that are ≥20 residues away in sequence but show PAE <6 to the target residue; we considered a segment of 5 residues to be disordered if the total number of “PAE neighbors” is ≤10. To identify helical linkers between domains, we defined secondary structure elements in AF models: ≥3 consecutive residues annotated as B or E by DSSP (Kabsch and Sander 1983) were considered as a beta-strand, and ≥6 consecutive residues annotated as G or H or I were considered as an alpha helix. We identified residues aligned to “acceptable HHsuite hits” or “acceptable Dali hits” in ECOD, and we dubbed them “candidate intra-domain residues.” We modified the above procedure for disorder region prediction to identify flexible helical linkers between domains by excluding the “PAE neighbors” from the same secondary structure elements and requiring the fraction of “candidate intra-domain residues” to be no more than 40%.

We partitioned each protein into non-overlapping five-residue segments and excluded segments with ≥3 residues from disordered regions or helical inter-domain linkers. We computed the average “same-domain” probability, i.e. Psame for every pair of segments. We sorted segment pairs reversely by Psame, and only considered pairs with Psame greater than x, a parameter to be optimized. The top-ranking segment pair initiated the first group. Starting from the second pair, we identified existing groups containing segments from this pair and handled three possible scenarios. First, when neither segment was included in existing groups, we created a new group. Second, when only one segment in this pair was included in an existing group, we merged the other segment into that group if the ratio between the average Psame for segments within the group, and the average Psame between the new segment and existing segments in the group was less than y, another parameter to be optimized; otherwise, the other segment initiated a new group. Third, if the two segments in this pair were present in two previously defined groups, we computed ${\mathrm{Psame}}^{1}$, ${\mathrm{Psame}}^{2}$, and ${\mathrm{Psame}}^{12}$, i.e. the average Psame within the first group, within the second group, and between the two groups. We merged the two groups if ${\mathrm{Psame}}^{1}<y\cdot {\mathrm{Psame}}^{12}$ or ${\mathrm{Psame}}^{2}<y\cdot {\mathrm{Psame}}^{12}$. In the above procedure, we optimized the two parameters, x and y, to maximize the percentage of correctly predicted domains (overlap between the true and predicted domains > 75% of residues in both). Through a grid search, we found the optimal values for x and y as 0.89 and 1.12.

Finally, we refined the boundaries of domains resulting from the clustering routine. We attempted to shorten or extend the domain boundaries based on each residue’s contacts, i.e. other residues <6 Å in 3D space but more than six residues away in sequence. If a residue at the domain boundary has more contacts out of this domain than within, we exclude it; in contrast, we add residues to extend domain boundaries if they have more contacts with residues within this domain than those outside it. After the boundary refinement, we kept domains with at least 20 residues.

## 3 Results

### 3.1 Input features and architecture of the DPAM-AI network

**Figure 1.**
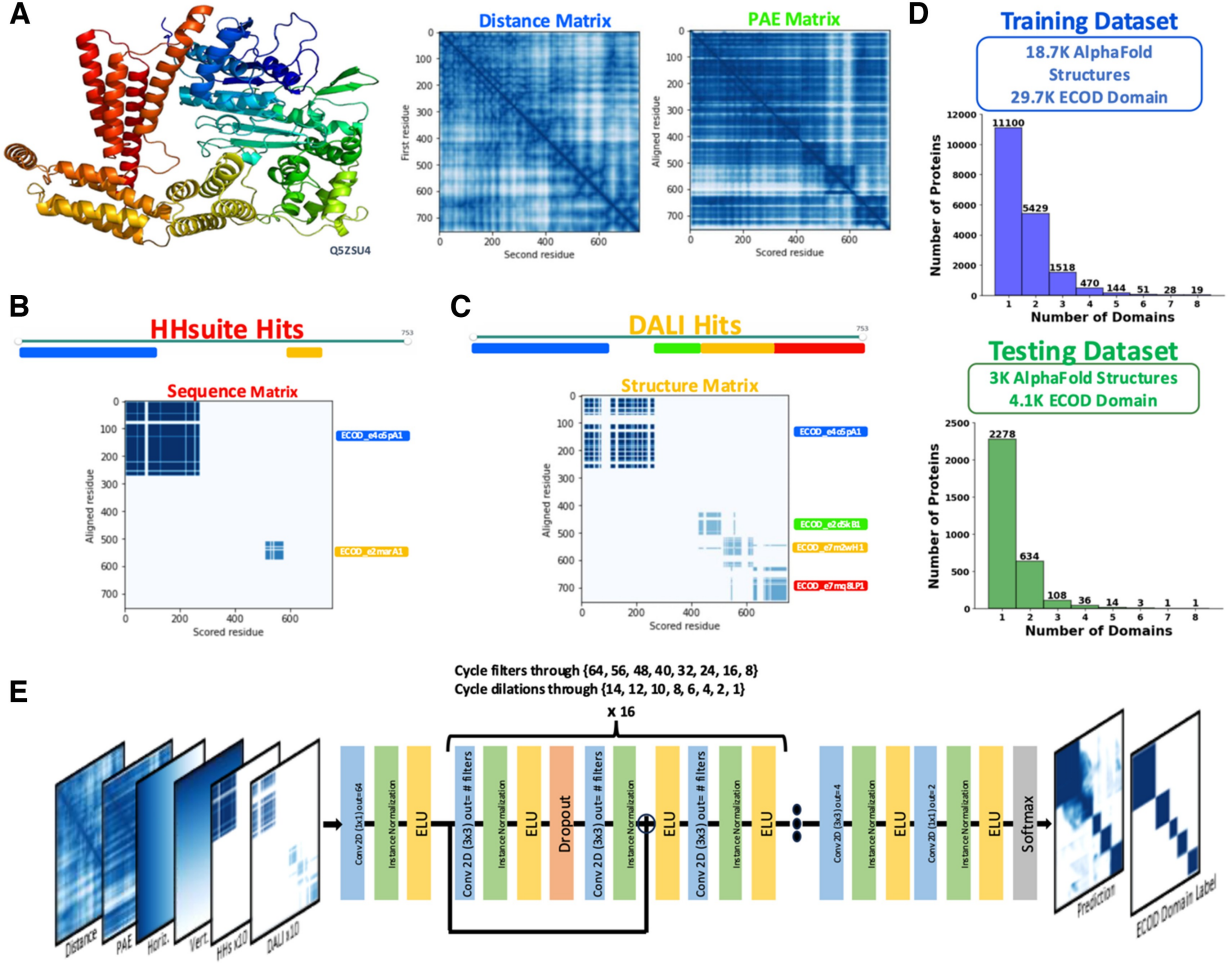
Input features and architecture of DPAM-AI. (A) AlphaFold model of Q5ZSU4 colored in rainbow (top), distance matrix to represent inter-residue distances in the 3D space (left), and PAE matrix to represent the estimated errors in the predicted distances between residues. (B) A 2D matrix to represent evidence derived from similar ECOD domains to the query protein found by Hhsuite sequence comparisons. (C) A 2D matrix to represent the evidence derived from similar ECOD domains to the query protein found by Dali 3D structure comparisons. (D) Histograms showing the distribution of domain numbers per protein in the training and testing datasets. (E) The DPAM-AI architecture.

Like DPAM, DPAM-AI uses 3D structure features and homology-based evidence to parse predicted structures into domains. The 3D features include distance and PAE matrices. As shown in Fig. 1A (left), the distance matrix represents the distances between residue pairs in the AlphaFold model, with shorter distances more likely to belong to the same domain. AlphaFold generates the PAE matrix (Fig. 1A, right), representing the estimated errors in the predicted inter-residue distances. Continuous segments of low inter-residue distance and low PAEs tend to correlate with globular domains. ECOD domains found by sequence (HHsuite, Fig. 1B) or structure (Dali, Fig. 1C) similarity searches are used as homology-based evidence. Query residues aligned to the same ECOD domain are more likely to belong to the same domain, and such information is represented as a series of 2D matrices, each labeling the residue pairs aligned to the same ECOD domain. Finally, we added two 2D matrices to store the residue numbers associated with each pixel in these 2D matrices. These 2D matrices, all indicative of the probability for residue pairs to be in the same domain, are stacked as different channels and used as the inputs for a CNN, which generates an output 2D matrix of the same shape.

The CNN was trained to predict whether pairs of residues are in the same domain based on ECOD domain definitions. The training and testing datasets consist of single- and multidomain proteins, but are dominated by multidomain proteins (Fig. 1D). We used 48 convolutional layers with residual blocks to reduce the problem of vanishing gradients. We also added 15 dropout layers (dropout ratio: 15%) to combat overfitting during network training (Fig. 1E). The convolutional layers progressively decrease in filter size and dilation rate after the initial projection into a higher filter dimensionality. Early layers with a high dilation rate allow each pixel in the later layers to capture broader contextual information. In contrast, the lower dilation rates for the later layers focus on local features. The decrease in filter size allows the network to gradually summarize the high-dimensional input features to the final softmax layer that outputs the predicted probabilities for residue pairs to be in the same domain, namely, same-domain probability. The 2D matrix with predicted same-domain probabilities resembles the 2D representation of ECOD domain definitions with some noise (Fig. 1E, right). Like DPAM (Zhang *et al.* 2023), we applied agglomerative clustering to group residues with high same-domain probabilities into domains.

### 3.2 Performance of DPAM-AI in detecting domains

**Figure 2.**
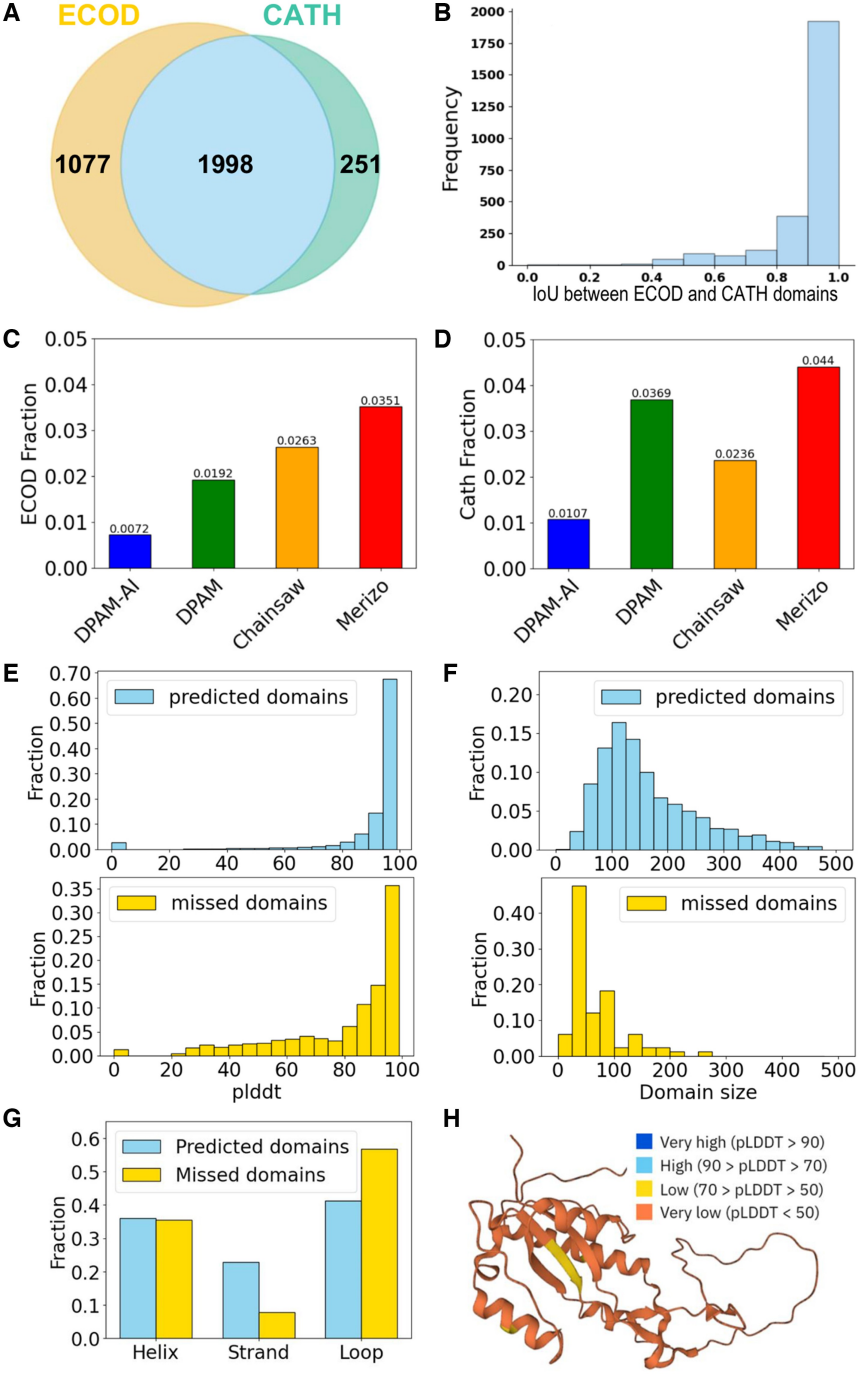
Performance of DPAM-AI in detecting domains from AlphaFold models. (A) Venn diagrams illustrating the overlap in proteins (PDB entries) included in test sets derived from ECOD and CATH, respectively. (B) The distribution of IoU scores between domains defined by ECOD and CATH for the shared set of proteins. Fraction of (C) ECOD and (D) CATH domains missed by different methods. (E) The distribution per-residue pLDDT values for ECOD domains detected (top) or missed (bottom) by DPAM-AI. (F) Domains missed by DPAM-AI tend to have smaller sizes (bottom) than those that are detected (top). (G) Domains missed by DPAM-AI tend to have a lower fraction of beta-strands and a higher fraction of loops. (H) A large domain (accession: Q44522) missed by DPAM-AI shows poor model quality (pLDDT).

We evaluated the performance of DPAM-AI against DPAM and two other recent AI-based domain parsers, Merizo and Chainsaw. We used two test datasets: ECOD (3075 proteins) and CATH (2249 proteins), with 1998 proteins common to both datasets (Fig. 2A). Domains defined by ECOD and CATH show high consistency, with most of them showing intersection-over-union (IoU) above 0.8 (Fig. 2B). Merizo and Chainsaw were trained with CATH domains, and thus, they are expected to perform better against the CATH benchmark, as opposed to DPAM and DPAM-AI, which were trained on ECOD domains.

We first evaluated whether automatic domain parsers could detect domains annotated by ECOD and CATH. We considered a reference domain to be detected by a parser if at least 50% of its residues were assigned to predicted domains. DPAM-AI was able to detect 99.3% of ECOD domains (Fig. 2C), outperforming DPAM (98.1%), Chainsaw (97.4%), and Merizo (96.5%). The performance of DPAM-AI is similar to the CATH benchmark (Fig. 2D). DPAM-AI detected 98.9% of CATH domains, again outperforming the other tools. To evaluate how frequently a reference ECOD or CATH domain was split into multiple predicted domains, we counted the number of predicted domains that overlap with this reference domain in >50% of its residues. We found that DPAM-AI never split a single domain into multiple for the entire test set; DPAM and Merizo only split domains in a few cases (∼0.1%), while Chainsaw split 8% of reference domains into multiple predicted domains.

In contrast to Merizo and Chainsaw, DPAM and DPAM-AI use additional homology-based evidence against the target domain databases (close hits removed to avoid information leakage), which likely contributes to their superior performance. A drawback of using such evidence is that homology searches are time-consuming. These searches are ultimately helpful for assigning the parsed domains to an evolutionary classification.

We analyzed the features of domains that DPAM-AI misses. Residues in the missed domains tend to have lower pLDDT (Fig. 2E), indicating worse 3D model quality. This is likely related to the fact that we used the error estimate, PAE, in domain parsing, and poorly modeled regions tend to display high PAE relative to other regions in a protein. Domains missed by DPAM-AI tend to be smaller (Fig. 2F) and have a lower fraction of beta-strands but a higher fraction of coils and loops (Fig. 2G). Smaller domains consisting of helices and loops, such as zinc fingers, tend to be loosely packed and adopt flexible 3D structures, making them harder to detect by structure features that favor compact domains. Detecting homology between such small domains might also be challenging (Krishna *et al.* 2003). An example of a large domain missed by DPAM-AI is shown in Fig. 2H, and the entire domain displays poor quality (pLDDT < 50).

### 3.3 Performance of DPAM-AI in delineating domain boundaries

**Figure 3.**
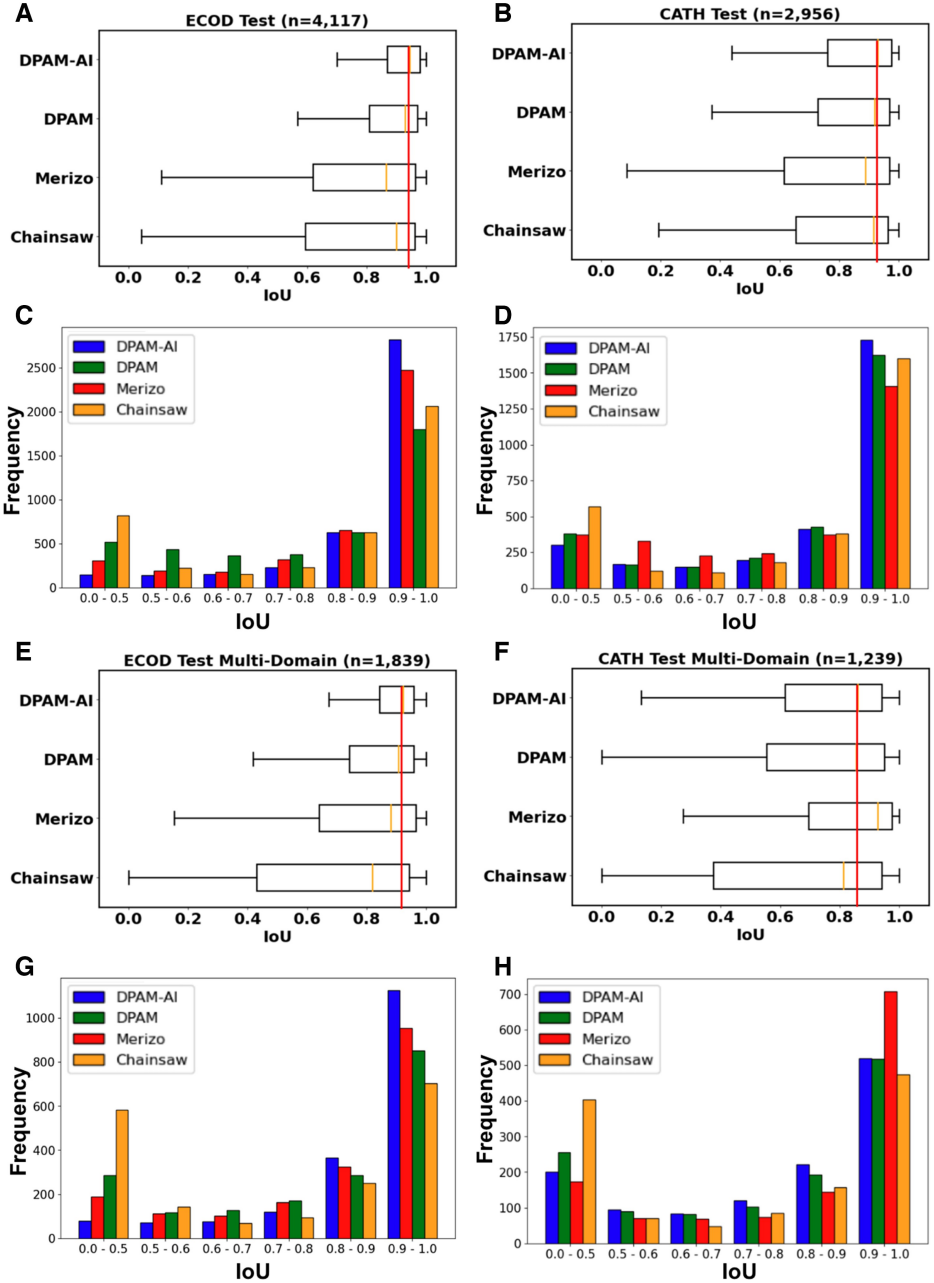
Performance of DPAM-AI in delineating domain boundaries. Box plots on top and bar graphs on the bottom show the distribution of IoU scores for different methods on (A, C) the ECOD test set, (B, D) the CATH test set, (E, G) the ECOD multidomain subset, and (F, H) the CATH multidomain subset.

To evaluate the prediction accuracy of domain boundaries, we identified the corresponding predicted domain showing the largest overlap with each reference domain in the ECOD test set. We evaluated the accuracy of domain boundaries by IoU, the intersection between residues in the reference domain and the predicted domain, divided by the union of the two sets. The distribution of IoU for the different methods is shown in Fig. 3A and C. DPAM-AI shows a median IoU of 0.95, outperforming DPAM (0.93), Chainsaw (0.91), and Merizo (0.87). Eighty-four percent of domains predicted by DPAM-AI show high agreement with ECOD’s definition with IoU above 0.8, higher than the other three methods. Over half of proteins in the ECOD test set consist of single domains, but it is more important to segment proteins with multiple domains correctly. On multidomain proteins, DPAM-AI, DPAM, and Chainsaw show slightly worse performance with median IoU of 0.92, 0.91, and 0.82. Surprisingly, Merizo’s performance (median IoU 0.88) on the multidomain ECOD test set is comparable to the entire set but still worse than DPAM or DPAM-AI (Fig. 3E and G).

We repeated this evaluation on a set of domains defined by CATH from proteins sharing no homology to our training dataset. Without explicit training on CATH domains, DPAM-AI still slightly outperforms all other methods on the CATH test set (Fig. 3B and D). Interestingly, although Merizo performs worst on the ECOD test set and single-domain proteins in the CATH test set, it is the best method for parsing multidomain proteins in the CATH test set (Fig. 3F and H). Merizo was trained with CATH domains and may be optimized to segment multidomain proteins. While Merizo is adapted to defining domains in agreement with CATH, its performance is not as generalizable to ECOD domains as Chainsaw, which was also trained on CATH domains.

**Figure 4.**
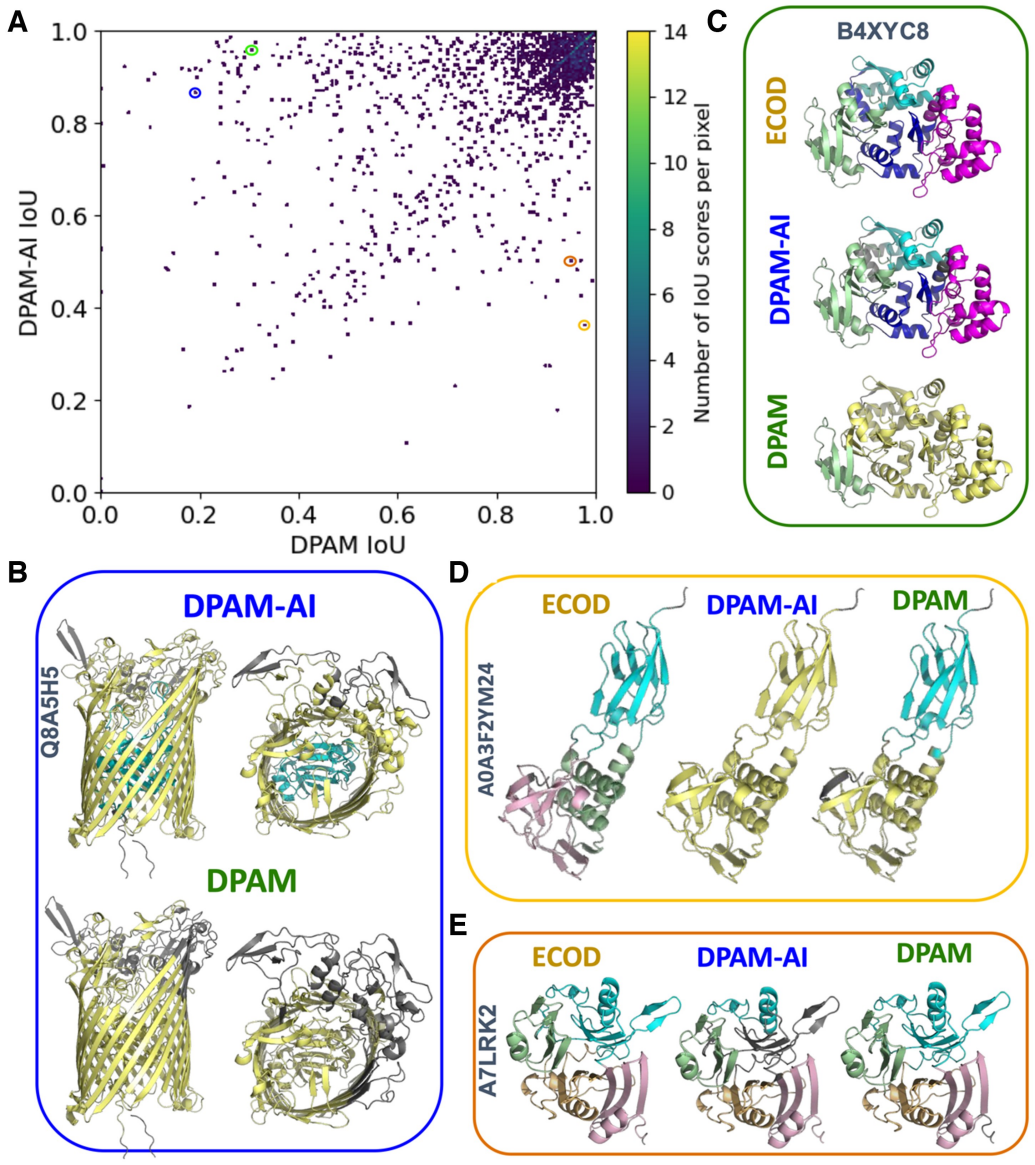
Comparison of parsed domains by DPAM and DPAM-AI. (A) A scatter plot showing the performance (measured by IoU) of DPAM-AI and DPAM on multidomain proteins from the ECOD test set. Each dot represents one ECOD domain, and the circled dots correspond to examples in (B-D). (B) An example where DPAM-AI correctly detects an additional domain (cyan) inside a β-barrel while DPAM fails. (C) DPAM-AI correctly separates multiple tightly packed and duplicated domains (cyan, blue, and magenta), while DPAM fails. (D) An example of a domain (cyan) with multiple discontinuous segments, for which DPAM-AI fails to recognize. (E) An example of a domain (cyan) with multiple segments discontinuous in sequence, for which DAPM-AI misses one segment (gray).

DPAM and DPAM-AI rely on similar input features, and the superior performance of DPAM-AI is primarily due to the CNN, which is more capable of integrating these features to predict the probabilities for residues to be in the same domain. To determine the causes of boundary differences between domains parsed by DPAM and DPAM-AI, we manually studied proteins in the ECOD multidomain test set for which the two methods showed substantial (> 0.1) IoU differences (Fig. 4A). Among the 1839 domains from the 797 multidomain proteins in this set, DPAM-AI performed substantially (ΔIoU > 0.1) better on 421 domains and substantially (ΔIoU < −0.1) worse on 156 domains. We found that DPAM-AI is better at splitting tightly packed domains (Fig. 4B) and segmenting domains composed of repeating units into single evolutionary units (Fig. 4C). However, DPAM-AI struggles more than DPAM to parse domains of discontinuous segments: 35.3% of the domains where DPAM-AI performed worse (ΔIoU < −0.1) were discontinuous. This fraction in the set where DPAM-AI performed better (ΔIoU > 0.1) was 12.6%. Two examples of discontinuous domains missed by DPAM-AI but correctly predicted by DPAM are shown in Fig. 4D and E. DPAM-AI’s inability to parse discontinuous domains likely stems from the rarity of such domains in the training data, and the AI model might have learned to disfavor them unless there is strong 3D structure or homology evidence.

### 3.4 Application of DPAM-AI to refine the boundaries of Pfam domains

3D structural data help delineate the boundaries and infer functions for domains detected through sequence comparisons. Such 3D information used to be solely available from experimental structures in the PDB. Among the 20 795 families in the Pfam database, 11 505 (55%) were linked to PDB entries. To expand the 3D structural coverage for Pfam families, we selected up to three representative AlphaFold models from AFDB for each Pfam family. These AlphaFold models significantly increase the fraction of Pfam families with 3D structures, allowing us to link 19 906 (95.7%) families to predicted structures. The 889 families without predicted 3D structure mostly (872, 98.1%) originate from viral proteins excluded from AFDB.

**Figure 5.**
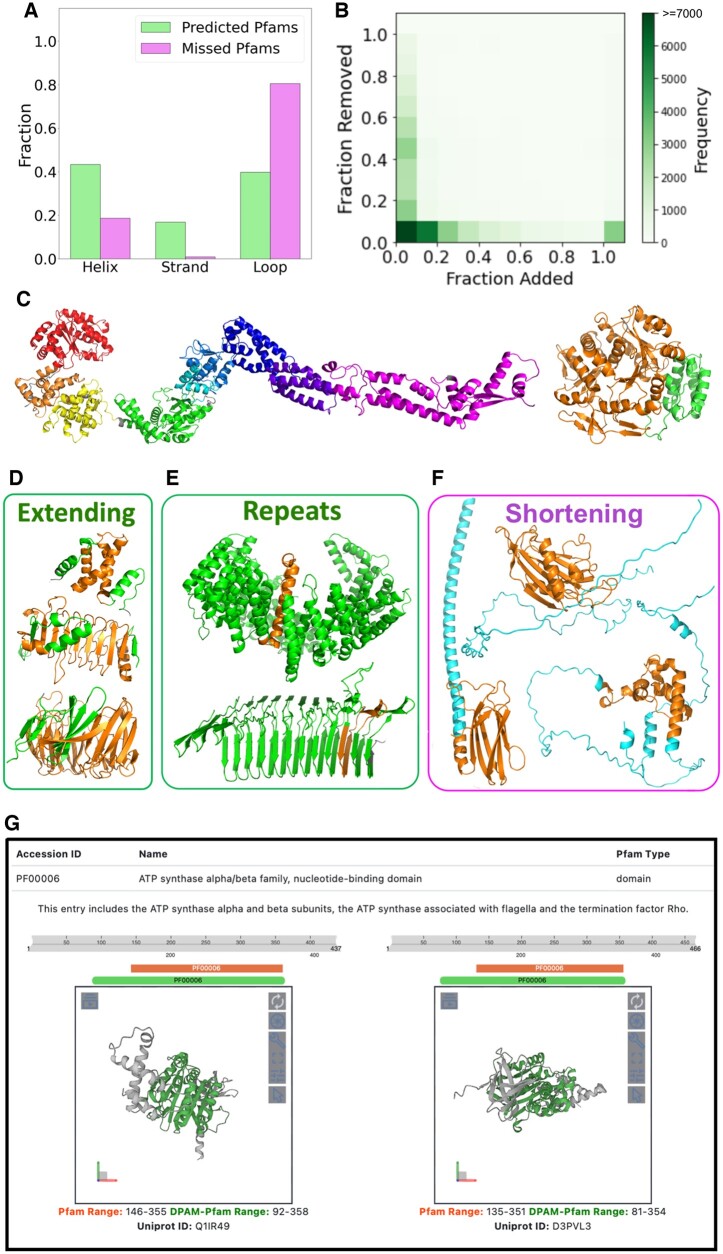
Comparison of domains defined by Pfam and DPAM-AI. (A) Pfam families without corresponding structural domains defined by DPAM-AI mostly consist of loops and are depleted of helices and strands. (B) Distribution (shown as heatmap) of the relative ratios (normalized by the length of the Pfam domains) of residues that are removed and added to the Pfam domains by DPAM-AI. If the relative ratio of added residues is above 1, we count it as 1. (C) Examples of Pfam domains that are split into multiple domains by DPAM-AI. Different DPAM-AI domains are shown in different colors. (D) Examples where DPAM-AI extends the boundaries of Pfam domains: residues belonging to the Pfam domains are in orange, and added residues are in green. (E) Repeats are the extreme cases where DPAM-AI significantly extends the Pfam domain boundary. (F) Examples where DPAM-AI shortens the boundaries of Pfam domains: residues belonging to the DPAM-AI domains are in orange, and residues in the Pfam domains but not DPAM-AI domains are in cyan. (G) A snapshot of our website to present the 3D structures and refined boundaries to Pfam domains.

We applied DPAM-AI to analyze 59 222 AlphaFold models representing 19 906 Pfam families. We detected DPAM-AI domains mapping to the same regions (overlap >50% of the Pfam or DPAM-AI domains) as the Pfam domains in these proteins. We found matching DPAM-AI domains for 52 673 Pfam domains representing 18 217 (91.5%) Pfam families. We compared the Pfam families with matching structural domains against those without matches; the latter tend to be shorter (213 versus 116) and contain fewer alpha helices or beta-strands (10.4 versus 1.5). Pfam domains without matching DPAM-AI domains are remarkably depleted of both strands and helices (Fig. 5A), indicating that they do not adopt rigid 3D structures.

Among the 52 673 Pfam domains, 8127 (15.4%) were split into multiple DPAM-AI domains, and examples of such cases are shown in Fig. 5C. The multiple DPAM-AI domains mapping to these Pfam families might be connected via relatively flexible linkers (Fig. 5C, left) or tightly packed against each other (Fig. 5C, right). While keeping them in single Pfam domains is helpful for functional inference, partitioning them into different structural domains allows for investigating other contexts where these domains may function and detecting distant evolutionary relationships.

The remaining 44 546 Pfam domains were mapped to single DPAM-AI domains, and the latter refined the boundary for a considerable fraction of Pfam domains. We calculated the ratio of residues added and removed by DPAM-AI, normalized by the length of Pfam domains. Only 36.1% (16 069 out of 44 546) of the DPAM-AI domains have similar boundaries as the Pfam domains, for which the percentage of removed or added residues is less than 10% of all residues in the Pfam domains (Fig. 5B). DPAM-AI extends the boundaries substantially (added residues >10%) for 45.5% of Pfam domains, and such extended regions frequently include additional secondary structure elements that are tightly packed against the Pfam domains (Fig. 5D). The most extreme cases of extending the Pfam boundaries are domains made of short repeats (Fig. 5E): while Pfam defines the minimal repeat units, DPAM-AI tends to delineate globular domains consisting of many tandem repeats. Meanwhile, DPAM-AI has removed more than 10% of residues for 24.7% of Pfam domains. As shown in Fig. 5F, the removed residues are frequently flexible helices and loops around the structural core. We presented the corresponding DPAM-AI domains for the Pfam domains as a public website at http://prodata.swmed.edu/DPAM-pfam/, allowing users to visualize the refined boundaries and 3D structures of Pfam domains (Fig. 5G).

We downloaded the Pfam seed alignments and the Pfam annotations of Uniprot entries (release 36) from the Pfam ftp site at https://ftp.ebi.ac.uk/pub/databases/Pfam/current_release/. For each Pfam family, we identified proteins containing this family’s domains and AlphaFold models in AFDB. We selected three (if available) representative AlphaFold models for each family. We favored proteins in the Pfam seed alignment and those with few inserted or deleted residues compared to the Pfam Hidden Markov Models. We used the DPAM-AI to predict domains from each selected AlphaFold model and identified DPAM-AI domains whose overlap with the Pfam domain in the model was larger than 50% of the Pfam domain length or 50% of the DPAM-AI domain length. We presented the Pfam domains and their corresponding DPAM-AI domains in an online database, where nightingale (Salazar *et al.* 2023) was used to show the domain diagrams, and mol* (Sehnal *et al.* 2021) was used to display the 3D structures of the DPAM-AI domains.

## 4 Conclusions

We have developed DPAM-AI, which utilizes a CNN to parse AlphaFold models into domains by integrating structural and homology-based evidence. DPAM-AI outperformed DPAM and other recently published domain parsers (Merizo and Chainsaw) in domain identification and boundary delineation. Although DPAM-AI was developed based on ECOD, it can be generalized to other structure classifications. We expect this tool to improve the evolutionary classification of AlphaFold models and to allow the scientific community to benefit the most from these structural data. We have applied DPAM-AI to analyze Pfam domains, splitting a considerable fraction into multiple domains and refining the boundaries for half of them. These results are shared through a public website at http://prodata.swmed.edu/DPAM-pfam/.

The domains identified by DPAM-AI represent the fundamental evolutionary, structural, and functional units of proteins. Accurate domain identification allows for their classification within evolutionary hierarchies while also facilitating functional predictions based on homology. Additionally, defining precise domain boundaries within proteins supports experimental biologists in designing targeted constructs for various downstream applications, including functional characterization and drug development, thereby accelerating biomedical research.
